# Supplementary material for: Identification of lectin receptors for conserved SARS‐CoV‐2 glycosylation sites
Source: EMBO J. 2021 Aug 23;40(19):e108375. doi: 10.15252/embj.2021108375 (PMC8420505; doi:10.15252/embj.2021108375)
Supplement: Supplementary file 1 — Appendix [file EMBJ-40-e108375-s002.pdf]

Appendix

Table of content

Appendix Figure S1	page 2, 3
Appendix Figure S2	page 4, 5
Appendix Figure S3	page 6
Appendix Figure S4	page 7, 8

Appendix Figure S1

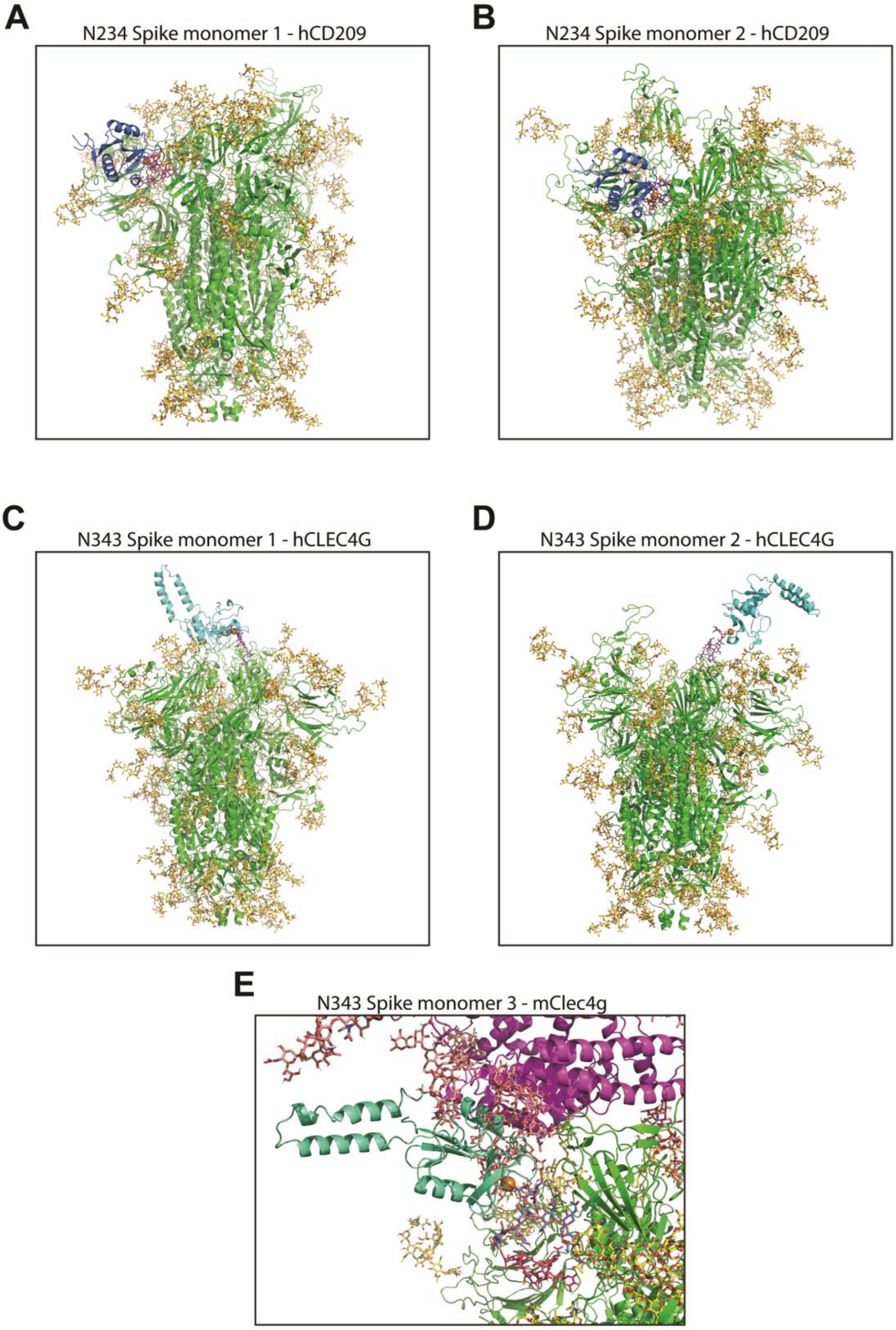

Appendix Figure S1. Structural modelling of lectin-Spike interactions.

(**A**, **B**) 3D structural modelling of glycosylated trimeric Spike (green with glycans in yellow) interacting with the CRD of hCD209 (dark blue with Ca^{2+} in orange). The model shows the (A) Spike monomer 1 and (B) Spike monomer 2 glycan site N234 (Oligomannose structure Man9 in red) bound to hCD209.

(**C**, **D**) 3D structural modelling of glycosylated trimeric Spike (green with glycans in yellow) interacting with the CRD of hCLEC4g (cyan with Ca^{2+} in orange). The model shows Spike (A) monomer 1 and (B) monomer 2 glycan site N343 (complex type glycan with terminal GlcNAc in purple-blue) bound to hCLEC4g.

(**E**) 3D structural modelling of glycosylated trimeric Spike (green with glycans in yellow) interacting with glycosylated human ACE2 (purple with glycans in salmon). The CRD of mClec4g (cyan with Ca^{2+} in orange) was modelled onto Spike monomer 3 glycan site N343 (complex type glycan with terminal GlcNAc in purple-blue). Structural superposition of mClec4g and ACE2 highlights steric incompatibility.

Appendix Figure S2

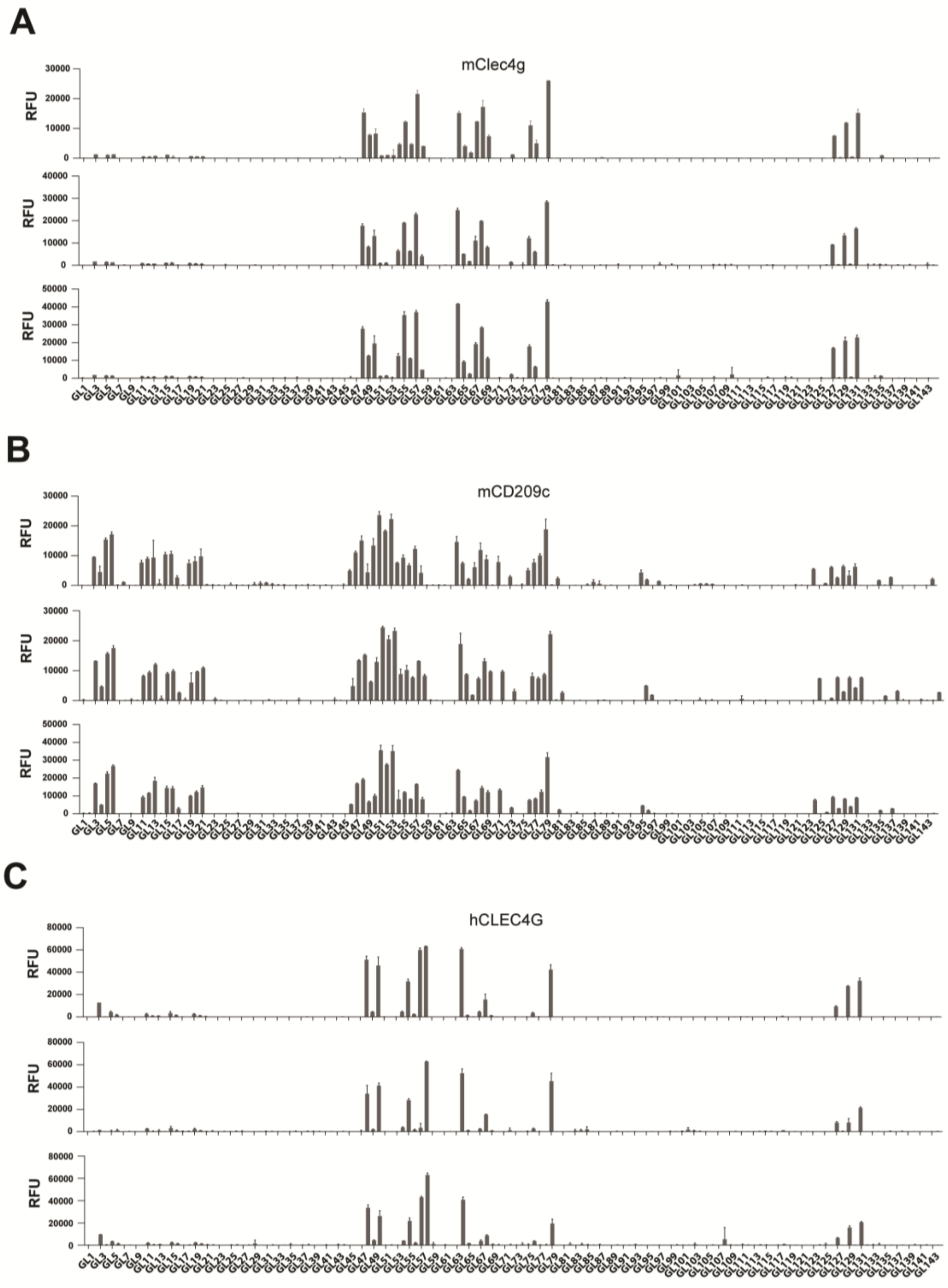

Appendix Figure S2. Glycan microarray results.

(A-C) Binding of mClec4g, mCD209c and hCLEC4G to glycans spotted on the microarray. Primary data from three independent microarray measurements are shown. Each histogram shows the average values of relative fluorescence units (RFU) +/- standard deviation (Technical replicates, N=4). Glycan structures of GL1 to GL144 is represented in Appendix Fig S3.

Appendix Figure S3.

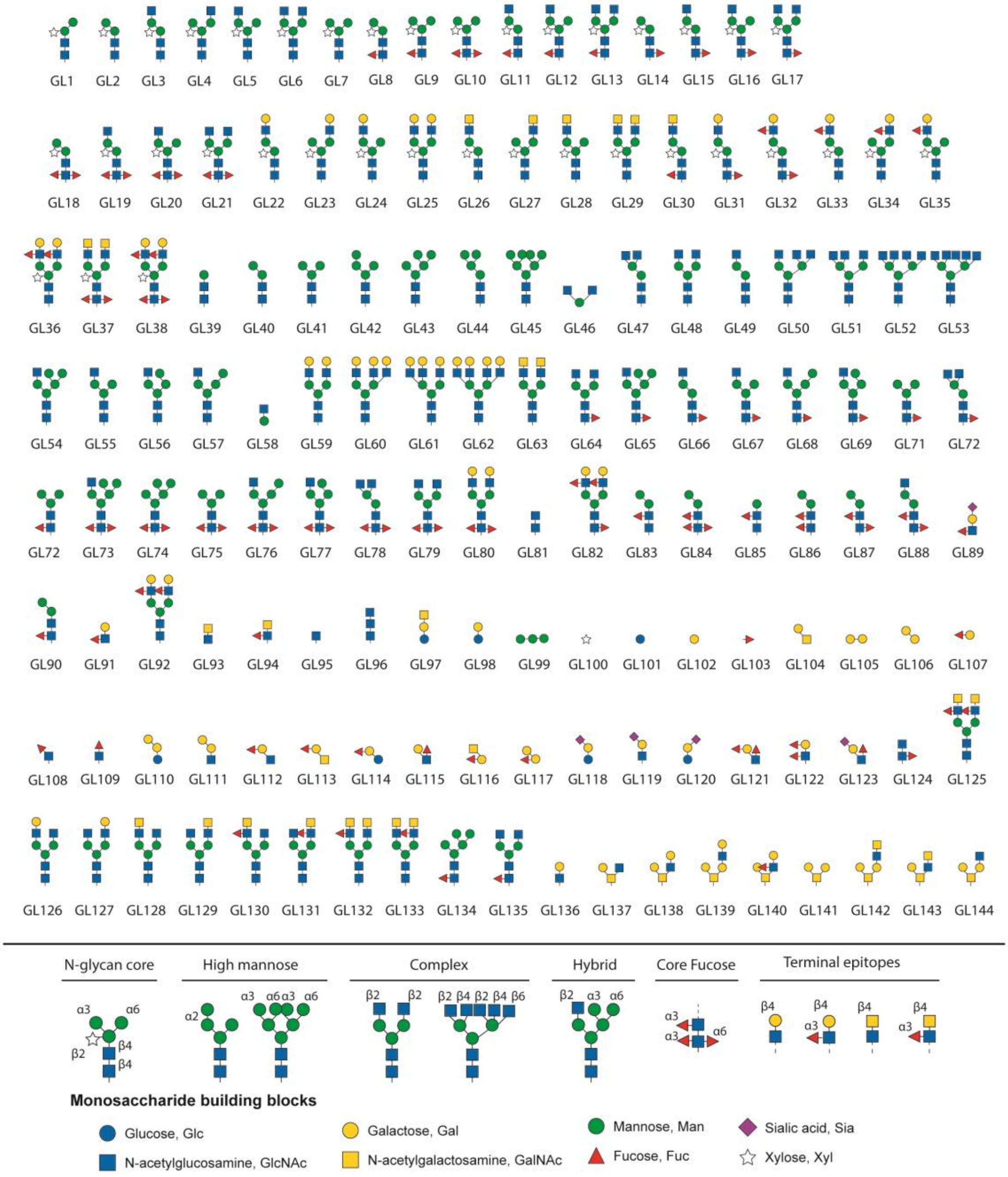

Appendix Figure S3. Schematic representation of the Glycan microarray.

Appendix Figure S4.

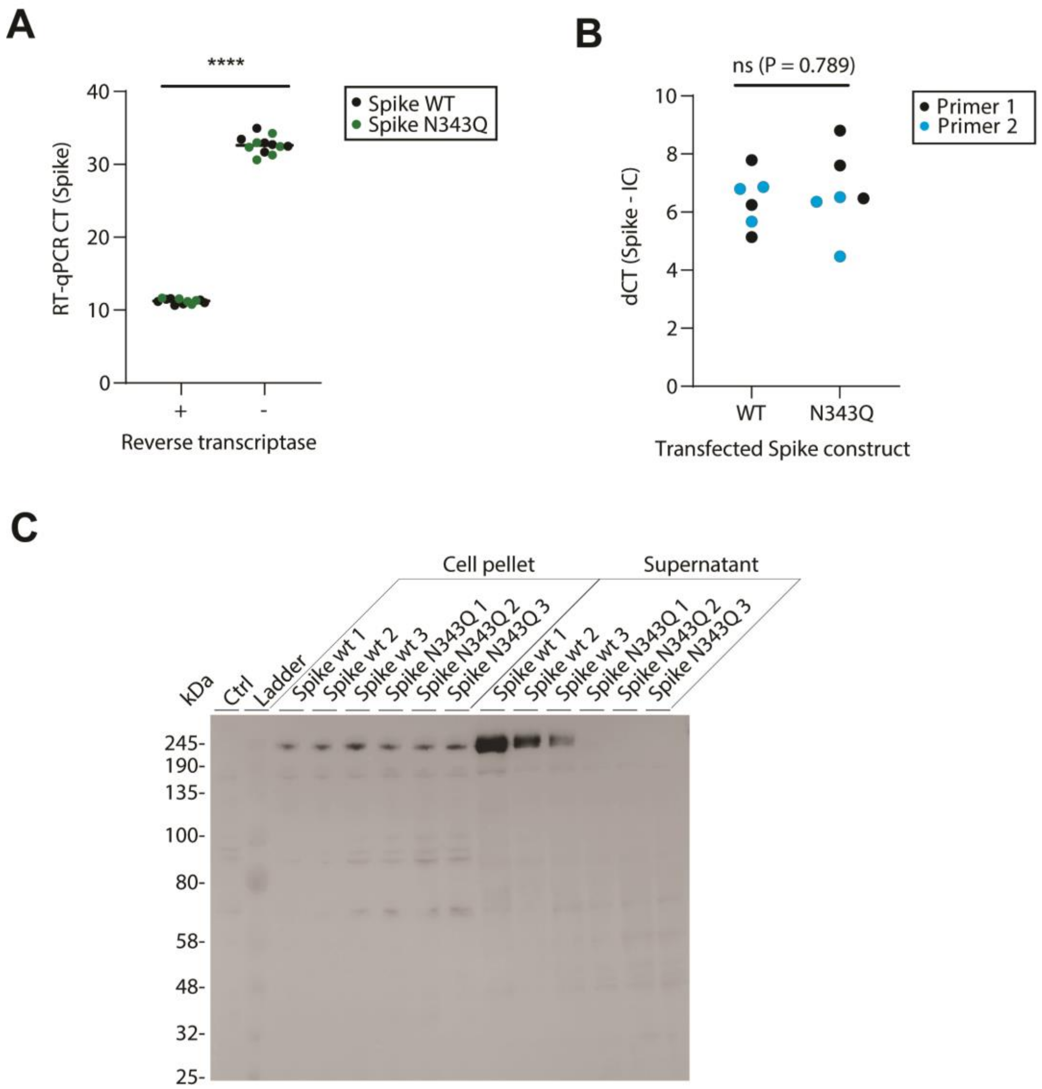

Appendix Figure S4. HEK293-6E cells transfection with wild-type or N343Q full-length Spike.

(**A**) Cycle-Threshold (CT) obtained in the presence or absence of reverse transcriptase (RT) during cDNA synthesis. Green and black dots mark each replicate of the two different constructs transfected.

(**B**) Adjusted Cycle-Threshold (delta CT, dCT) for each replicate, adjusted to an internal control gene (eIF2B2). Blue and black dots mark replicates from two different qPCR primer sets to detect Spike RNA.

(C) Spike protein were detected by western blot using His-tag-specific antibody. Protein extracts from cell pellets or supernatants from wt and N343Q Spike of three biologic replicates were loaded. Ctrl, lysate of untransfected HEK293-6E cells.

Data information: For both (A) and (B), transfections were performed in three biologic replicates. Student t-test; ****P<0.0001.
