# Supplementary material for: Identification of lectin receptors for conserved SARS‐CoV‐2 glycosylation sites
Source: EMBO J. 2021 Aug 23;40(19):e108375. doi: 10.15252/embj.2021108375 (PMC8420505; doi:10.15252/embj.2021108375)
Supplement: Supplementary file 2 — Expanded View Figures PDF [file EMBJ-40-e108375-s005.pdf]

Expanded View Figures

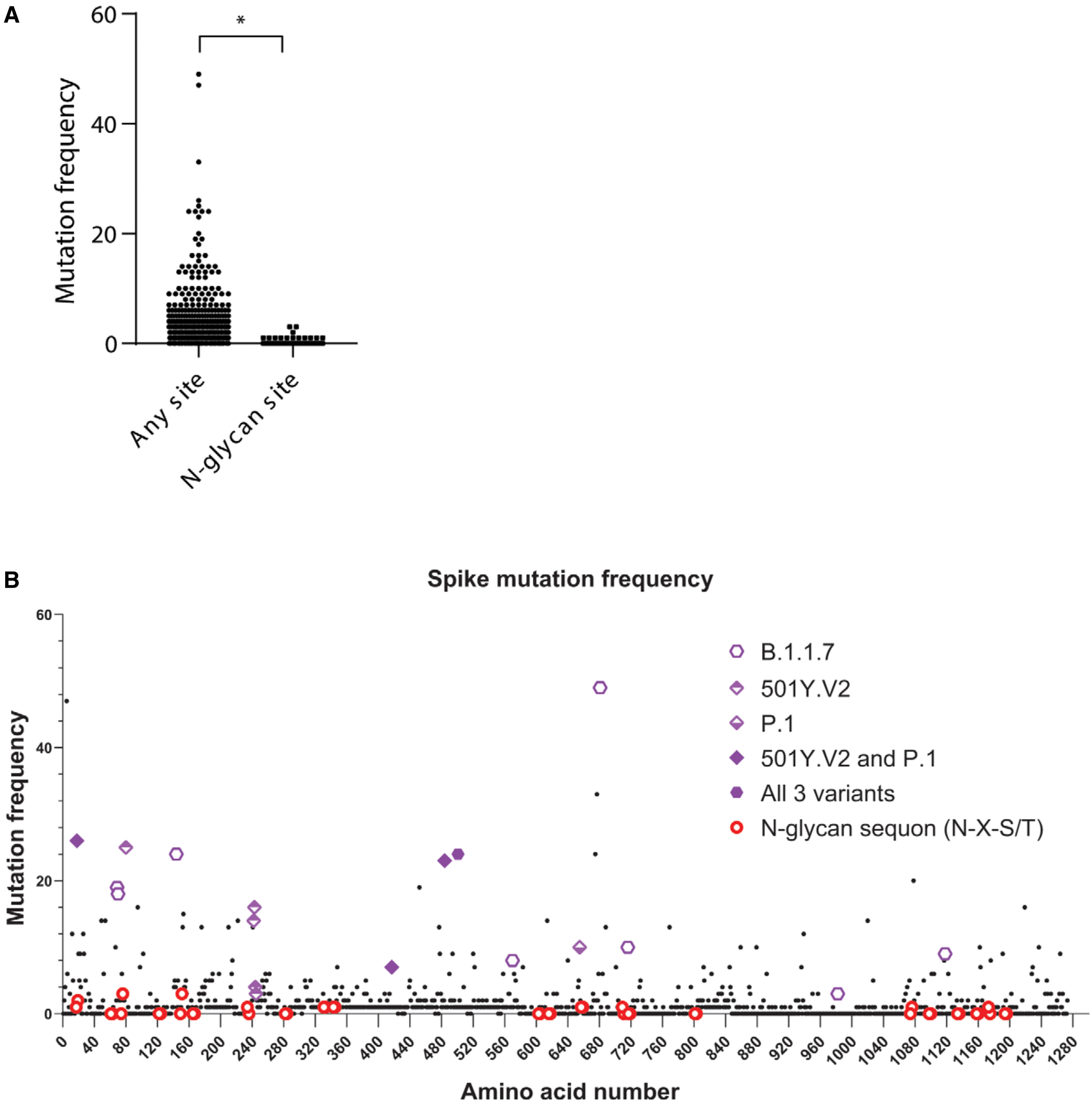

Figure EV1. Mutation frequency of *N*-glycan sites on SARS-CoV-2 Spike.

A Among the 1,273 amino acids of Spike the frequency of mutational amino acid conversion within N-glycan sequons is plotted against all other sites.

B The mutation frequency of all 1,273 amino acids of Spike is shown. *N*-glycan sequons and mutations harboured by the new variants B.1.1.7, 501Y.V2 and P.1 are highlighted.

 © 2021 The Authors

Data information: (A) Two-tailed Student's *t*-test, $*P < 0.05$.

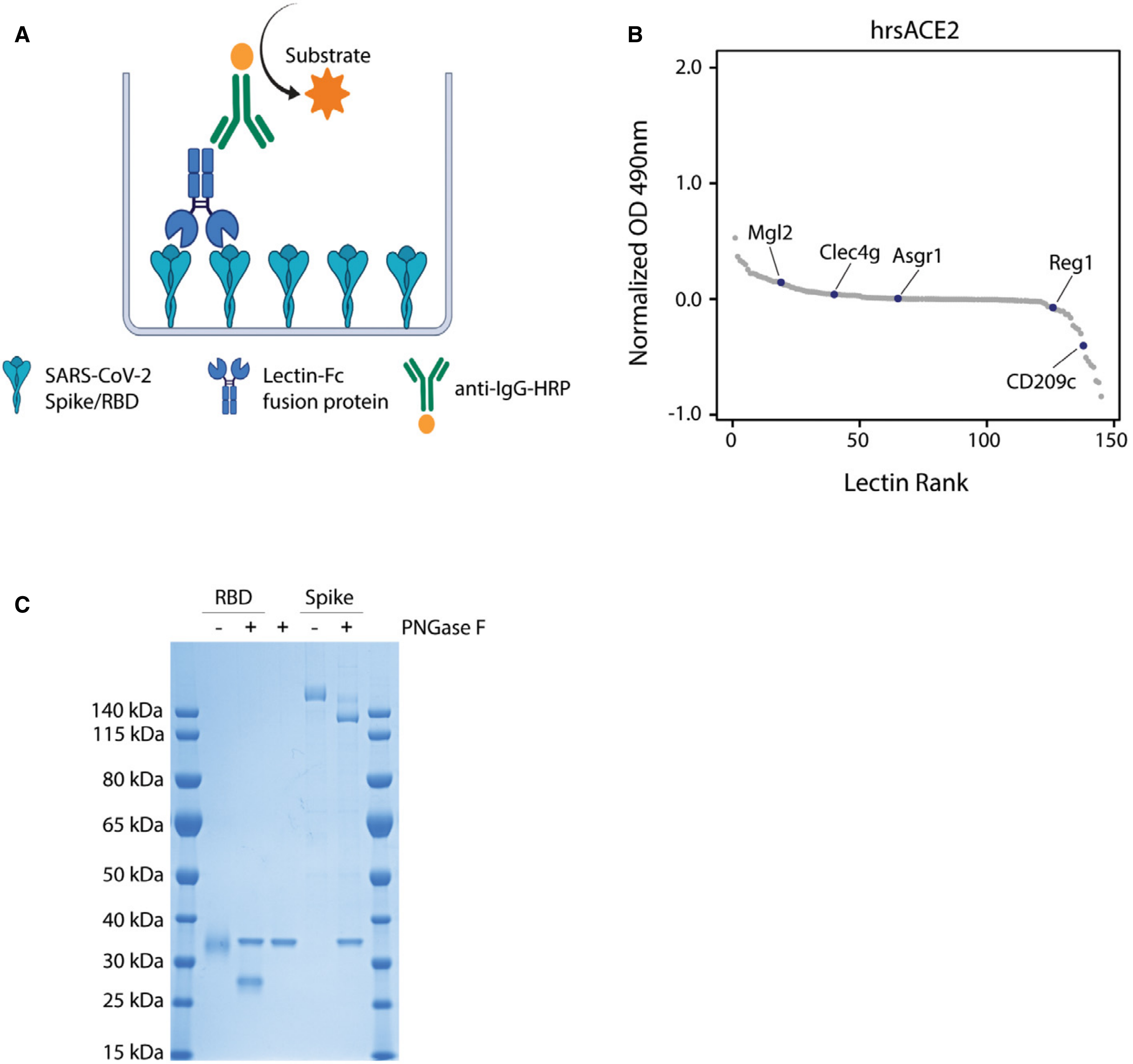

Figure EV2. ELISA assays to detect lectin binding.

A Schematic representation of the ELISA protocol, consisting of coating with trimeric full-length Spike or the monomeric receptor binding domain (RBD) followed by sequential incubation with lectin-Fc fusion proteins and secondary anti-IgG-HRP antibodies. The binding of lectin-Fc fusion proteins was quantified by peroxidase-dependent substrate conversion, measured by optical density (OD) at 490 nm and normalized against a BSA control.

B ELISA screen of the lectin-Fc library against human recombinant soluble ACE2 (hrsACE2). Results are shown as mean OD values of 2 replicates normalized against a BSA control and ranked by value. The 5 main lectin targets identified by Spike and RBD ELISA screen (Fig 2A and B) are labelled.

C SDS–Page of RBD and full-length trimeric Spike de-*N*-glycosylated with PNGase F and stained with Coomassie blue. A PNGase F control was added to display the size of the PNGase F protein.

© 2021 The Authors

Figure EV3. Single-molecule atomic force microscopy of a single trimeric Spike binding to murine Clec4g or CD209c.

A, B Unbinding forces versus loading rates for trimeric Spike dissociating from (A) Clec4g-Fc or (B) CD209c-Fc. Unbinding forces were determined from the magnitude of the vertical jumps measured during pulling of the cantilever (Fig 3B) and individually plotted versus the respective force loading rates (equal to the pulling speed times effective spring constant) to decipher the dissociation dynamics (Table 1). A well-defined single-bond behaviour of a unique monovalent bond was found (red dots) that, in line with Evans's single energy barrier model, yielded a linear rise of the unbinding force with respect to a logarithmically increasing loading rates for both (A) Clec4g and (B) CD209c. Double (green) and triple (blue) bond behaviours were calculated according to the Markov binding model using parameters derived from the single barrier model. Unbinding force values scattered between single and triple bond strengths, indicating that interactions with various glycosylation sites with different binding strengths. pN = picoNewton, pN/s = picoNewton per second.

C–F High-speed AFM of a single trimeric Spike visualizing the real-time interaction dynamics with lectins. (C) 5 frames of Clec4g or CD209c alone imaged on mica. (D) Volume distribution of single trimeric Spike (2,737 ± 65 nm^3, N = 6), trimeric Spike/Clec4g (4,005 ± 417 nm^3, N = 27) and trimeric Spike/CD209c complexes (5,034 ± 364 nm^3, N = 24), as well as numbers of lectins bound to trimeric Spike, averaged over the experimental recording period ± SD (all technical replicates). The colours indicate the range of bound lectins, for Clec4g/trimeric Spike (3.2 ± 1.1) and for CD209c/trimeric Spike (5.2 ± 1.0). (E) Sequential movie frames of trimeric Spike/Clec4g complexes, acquired at a rate of 153.6 ms/frame, corresponding to Fig 3E. (F) Sequential movie frames of trimeric Spike/CD209c complexes, acquired at 303 ms/frame, corresponding to Fig 3E. White arrows point to lectins associating with the Spike trimer body. Red arrows indicate dissociation of lectins from the Spike trimer, highlighting positions where the lectin was bound in the previous frame. Blue dotted ellipses display low mobility regions. Colour schemes indicate height in nanometers (nm).

 © 2021 The Authors

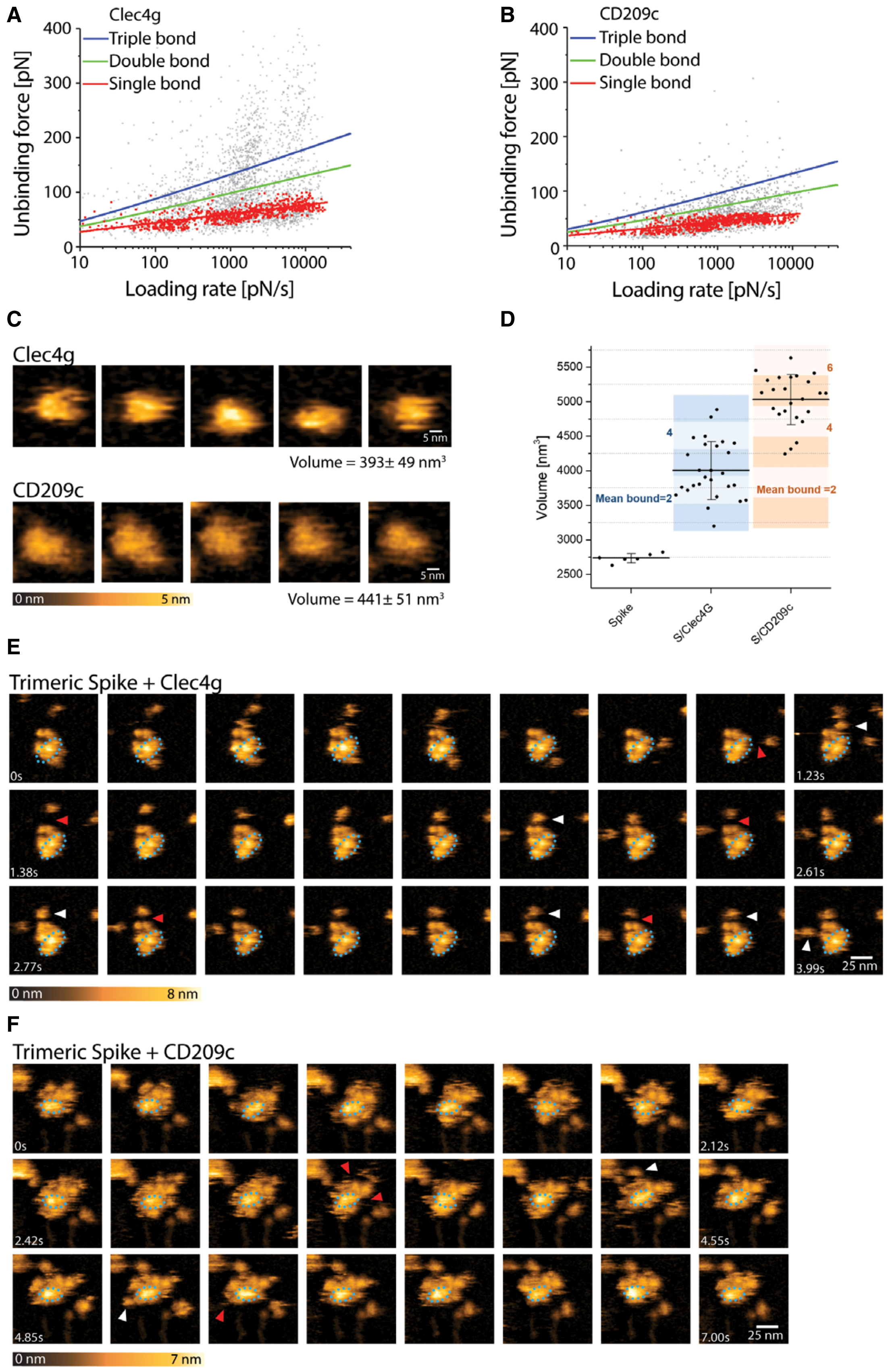
A
Clec4g
Triple bond
Double bond
Single bond
Unbinding force [pN]
Loading rate [pN/s]
B
CD209c
Triple bond
Double bond
Single bond
Unbinding force [pN]
Loading rate [pN/s]
C
Clec4g
5 nm
Volume = 393± 49 nm³
CD209c
5 nm
0 nm
5 nm
Volume = 441± 51 nm³
D
Volume [nm³]
Mean bound=2
Mean bound =2
Spike
S/Clec4G
S/CD209c
E
Trimeric Spike + Clec4g
0s
1.23s
1.38s
2.61s
2.77s
3.99s
25 nm
0 nm
8 nm
F
Trimeric Spike + CD209c
0s
2.12s
2.42s
4.55s
4.85s
7.00s
25 nm
0 nm
7 nm

Figure EV3.

Figure EV4. Single-molecule atomic force microscopy of a single trimeric Spike binding to human CLEC4g or CD209.

A Single-molecule force spectroscopy (SMFS) to determine the binding probability for trimeric Spike to mica coated hCLEC4G and hCD209. Data are shown as mean binding probabilities ± SD of single, double, triple or quadruple bonds (technical replicates, *N* = 4).

B, C Unbinding forces versus loading rates for a single trimeric Spike dissociating from (B) hCLEC4G or (C) hCD209. Unbinding forces were determined from the magnitude of the vertical jumps measured during pulling (Fig 3B) and individually plotted vs. their force loading rates (equal to the pulling speed times effective spring constant) to assess the dissociation dynamics (Table 1). Single-bond interactions (red dots) were fitted using the Bell-Evans single barrier model (red line). A well-defined single-bond behaviour of a unique monovalent bond was found (red dots) that, in line with Evans's single energy barrier model, yielded a linear rise of the unbinding force with respect to a logarithmically increasing loading rate for both (B) hCLEC4g and (C) hCD209. Double (green) and triple (blue) bond behaviours were calculated according to the Markov binding model using parameters derived from the single barrier model. Unbinding force values scattered between single and triple bond strengths, indicating that they arise from multiple interactions with various glycosylation sites. pN=picoNewton, pN/s = picoNewton per second.

D–G High-speed AFM of a single trimeric Spike visualizing the real-time interaction dynamics with lectins. (D) 5 frames of hCLEC4g or hCD209 alone imaged on mica. (E) Volume distributions of single trimeric Spike (2,737 ± 65 nm^3, *N* = 6), trimeric Spike/hClec4g (3,529 ± 182 nm^3, *N* = 21) and trimeric Spike/hCD209 (4,272 ± 621 nm^3, *N* = 21) complexes, as well as the numbers of lectins bound to trimeric Spike, averaged over the experimental recording time ± SD (all technical replicates). The colours indicate the range of bound lectins, for hClec4g/trimeric Spike (3.5 ± 1.1) and for hCD209/trimeric Spike (3.8 ± 1.6). (F) Sequential movie frames of trimeric Spike/hCLEC4g complexes, acquired at a rate of 303 ms/frame, corresponding to Fig 4E. (G) Sequential movie frames of trimeric Spike/hCD209 complexes, acquired at 153.6 ms/frame, corresponding to Fig 4E. White arrows point to lectins associating with the Spike trimer body. Red arrows indicate dissociation of lectins from the Spike trimer, highlighting positions where the lectin was bound in the previous frame. The blue dotted ellipses display low mobility regions. Colour schemes indicate height in nanometers (nm).

 © 2021 The Authors

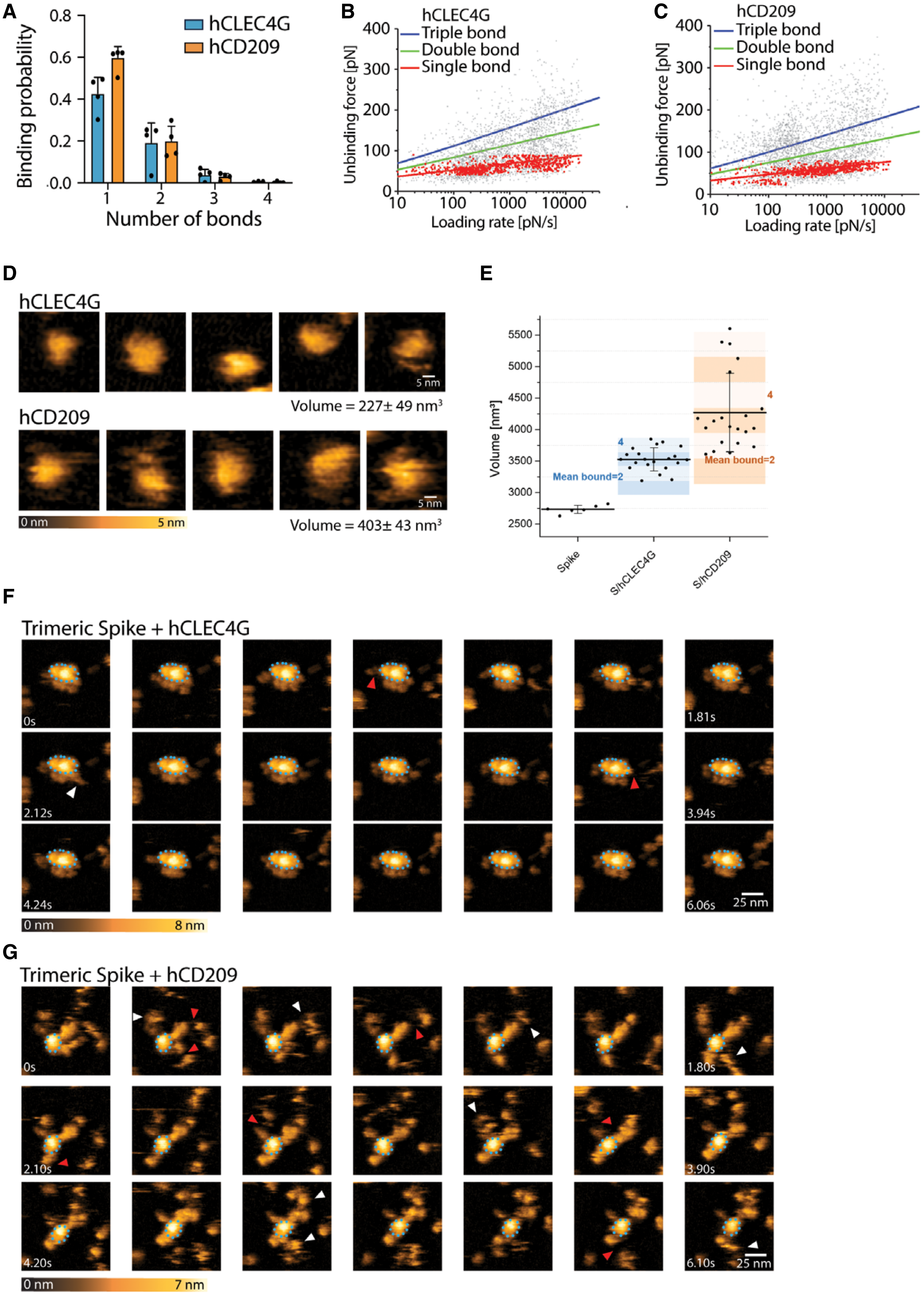
A
Binding probability
0.8
0.6
0.4
0.2
0.0
hCLEC4G
hCD209
1
2
3
4
Number of bonds
B
hCLEC4G
Triple bond
Double bond
Single bond
Unbinding force [pN]
Loading rate [pN/s]
C
hCD209
Triple bond
Double bond
Single bond
Unbinding force [pN]
Loading rate [pN/s]
D
hCLEC4G
5 nm
Volume = 227± 49 nm³
hCD209
5 nm
0 nm
5 nm
Volume = 403± 43 nm³
E
Volume [nm³]
Mean bound=2
Mean bound=2
Spike
S/hCLEC4G
S/hCD209
F
Trimeric Spike + hCLEC4G
0s
1.81s
2.12s
3.94s
4.24s
6.06s
25 nm
0 nm
8 nm
G
Trimeric Spike + hCD209
0s
1.80s
2.10s
3.90s
4.20s
6.10s
25 nm
0 nm
7 nm

Figure EV4.

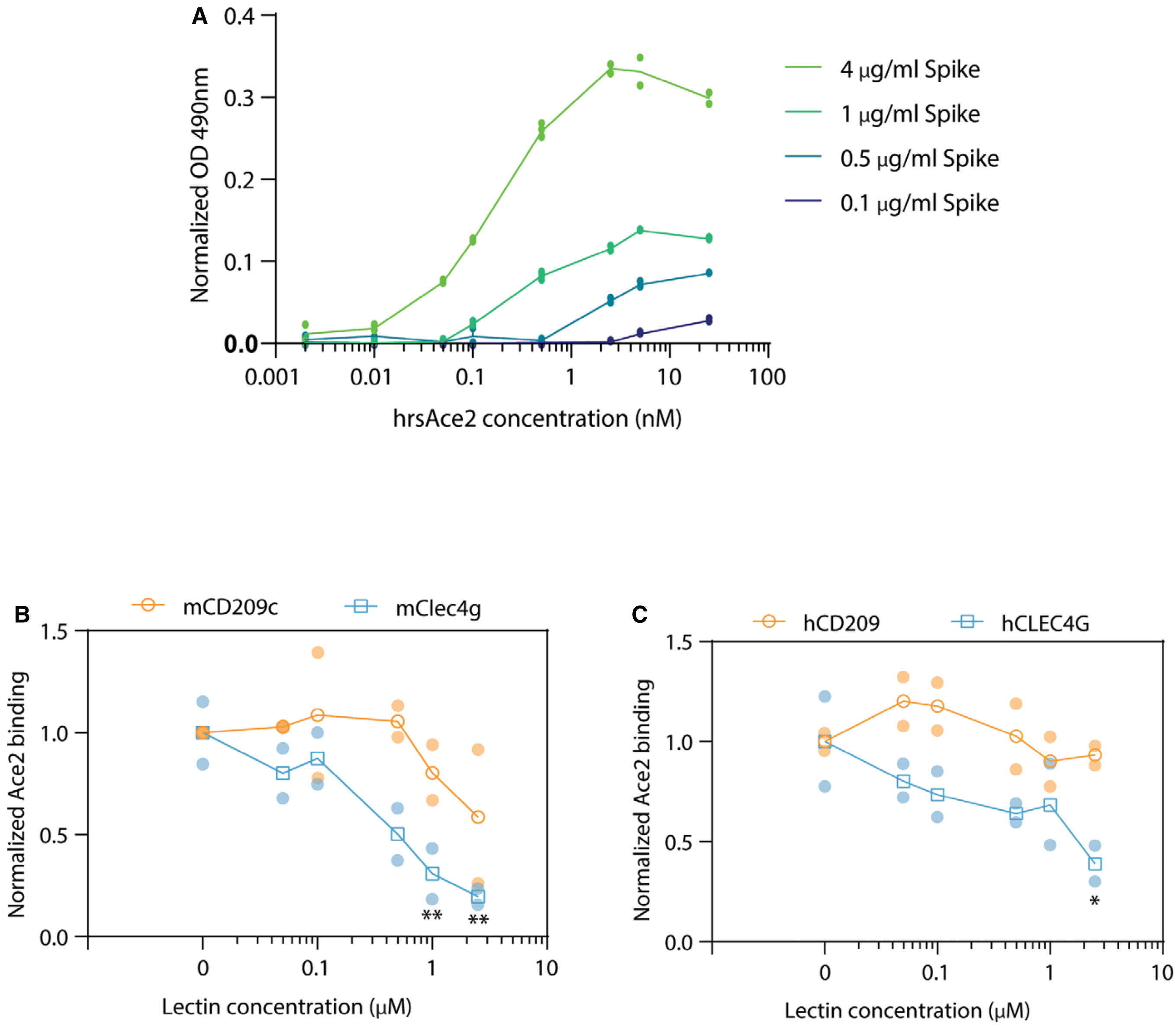

Figure EV5. Competition ELISA of ACE2 and Lectins for Spike binding.

A Titration of trimer Spike (4, 1, 0.5 or 0.1 μg/ml) and human recombinant soluble ACE2 (hrsACE2; 0.002, 0.01, 0.05, 0.1, 0.5, 2.5, 5, 25 nM) to define the optimal concentrations for the competition ELISA assay. Data are shown as average values ± standard deviation (Technical replicates, N = 2–4).

B Competition between 0.1 nM rhsACE2 and mCD209c or mClec4g (0, 0.05, 0.1, 0.5, 1, 2.5 μM) for binding of 4 μg/ml trimeric Spike coated to ELISA plates. Data are shown as mean OD normalized to 0 μM lectin condition.

C Competition between 0.1 nM rhsACE2 and hCD209 or hCLEC4G (0, 0.05, 0.1, 0.5, 1 and 2.5 μM) for binding to 4 μg/ml trimeric Spike coated to ELISA plates. Data is shown as mean OD normalized to the 0 μM lectin control.

Data information: For (B) and (C), each condition was measured in duplicates and shown as average values ± standard deviation. Two-way ANOVA with Sidak's multiple comparisons test; $*P < 0.05$ $**P < 0.01$.

© 2021 The Authors
