# Supplementary material for: Identification of lectin receptors for conserved SARS‐CoV‐2 glycosylation sites
Source: EMBO J. 2021 Aug 23;40(19):e108375. doi: 10.15252/embj.2021108375 (PMC8420505; doi:10.15252/embj.2021108375)
Supplement: Supplementary file 9 — Movie EV3 [file EMBJ-40-e108375-s003.zip › Movie EV3 legend.docx]

**Movie EV3.** High speed AFM of single trimeric Spike visualizing the real-time interaction dynamics with hCLEC4G acquired at a rate of 303 ms/frame.
